# Splice site SNPs of phospholipase PLCXD3 are significantly associated with variant and sporadic Creutzfeldt-Jakob disease

**DOI:** 10.1186/1471-2350-14-91

**Published:** 2013-09-12

**Authors:** Matthew T Bishop, Pascual Sanchez-Juan, Richard SG Knight

**Affiliations:** 1 National CJD Research & Surveillance Unit, University of Edinburgh, Western General Hospital, Crewe Road, Edinburgh EH4 2XU, UK; 2 Neurology Department, University Hospital “Marqués de Valdecilla”. Fundación “Marqués de Valdecilla” (University of Cantabria) IFIMAV and Centro de Investigación Biomédica en Red sobre Enfermedades Neurodegenerativas (CIBERNED), Santander, Spain

**Keywords:** Prion disease, Infection, Neurodegeneration, Susceptibility, Phospholipase

## Abstract

**Background:**

Variant Creutzfeldt-Jakob disease is an infectious, neurodegenerative, protein-misfolding disease, of the prion disease family, originally acquired through ingestion of meat products contaminated with bovine spongiform encephalopathy (BSE). Public health concern was increased by the discovery of human-to-human transmission via blood transfusion. This study has verified a novel genetic marker linked to disease risk.

**Methods:**

SNP imputation and association testing indicated those genes that had significant linkage to disease risk and one gene was investigated further with Sanger resequencing. Results from variant Creutzfeldt-Jakob disease were compared with those from sporadic (idiopathic) Creutzfeldt-Jakob disease and published controls.

**Results:**

The most significant disease risk, in addition to the prion protein gene, was for the phosphatidylinositol-specific phospholipase C, X domain containing 3 (PLCXD3) gene. Sanger resequencing of CJD patients across a region of PLCXD3 with known variants confirmed three SNPs associated with variant and sporadic CJD.

**Conclusions:**

These data provide the first highly significant confirmation of SNP allele frequencies for a novel CJD candidate gene providing new avenues for investigating these neurodegenerative prion diseases. The phospholipase PLCXD3 is primarily expressed in the brain and is associated with lipid catabolism and signal transduction.

## Background

Prion diseases are a family of fatal neurodegenerative diseases that can be idiopathic, acquired through infection, or associated with genetic mutations; Creutzfeldt-Jakob disease (CJD; OMIM 123400) being the main human disease. They are characterised by the tissue deposition of an abnormal misfolded form of the host-encoded prion protein (PrP), termed PrP^Sc^. The potentially infectious nature of prion disease makes it unique amongst other protein-misfolding neurodegenerative human diseases such as Alzheimer’s disease. In this study, patient cohorts were from the idiopathic sporadic CJD (sCJD) and from the acquired variant CJD. Sporadic CJD occurs worldwide with an annual mortality rate of approximately 1 per million per year, presenting typically as a rapidly progressive encephalopathy in the middle-aged and elderly, with conclusive diagnosis from neuropathological examination of the brain (showing spongiform change, gliosis, neuronal loss, and the hallmark deposition of PrP^Sc^). Variant CJD was first described in 1996, arising initially from food contamination with bovine spongiform encephalopathy (BSE) infectious material [1]. Variant CJD has since been diagnosed in 176 individuals from the United Kingdom and 51 from other countries, and has recently been associated with infection via blood transfusion. In relation to sCJD, variant CJD generally affects a younger age group, presents differently (with predominantly psychiatric early features) and with a slower clinical progression. Both sCJD and vCJD share similar pathological features representative of a prionopathy, however vCJD has some additional specific neuropathological features [2]. Prion protein gene (*PRNP*) analysis has shown that all examined definite and probable cases of vCJD (diagnostic criteria [http://www.cjd.ed.ac.uk/documents/criteria.pdf]) are methionine homozygous (MM) at codon 129 (dbSNP reference rs1799990). Sporadic CJD patients have an over-abundance of homozygotes for both alleles (59.5% MM, 21.4% MV, and 19.1% VV) when compared with UK genotype frequencies for controls (44.1% MM, 44.5% MV, and 13.4% VV) [3]. After a peak in 2000, the annual mortality rate for vCJD has since fallen, with total future cases at an estimated median level of 390 for the years 2010 to 2179 [4]. The potential for a silent epidemic of asymptomatic infection lead to tissue screening programs looking for PrP^Sc^ deposition in appendices or tonsils (presymptomatic lymphoreticular involvement is found in vCJD [5,6]). The most recent appendix survey indicates that 1 in 2000 individuals in the United Kingdom may have asymptomatic vCJD infection [7].

Identification of a genetic component to risk of vCJD infection may explain why relatively few people have died from vCJD despite significant dietary exposure in the UK [8] and could give an insight into the pathological processes specific to this disease. Currently the only non-*PRNP* gene targets found have limited confirmed associations to vCJD: cathepsin D [9], MTMR7 [10], and RARB and STMN2 [11]. The aim of this study was to take the genome-wide association (GWAS) data that we have previously published [10] and impute additional SNPs based on published genome genotype data to uncover further candidate genes which would then be resequenced in vCJD and sCJD populations for validation.

## Methods

### Imputation

Imputation was carried out using the following software:

•R programming environment (http://www.R-project.org)

•R package GenABEL (http://www.genabel.org) [12]

•minimac (http://genome.sph.umich.edu/wiki/Minimac) [13]

•MACH (Markov Chain based haplotyper; http://www.sph.umich.edu/csg/abecasis/MACH/index.html) [14]

•PLINK (Version 1.07-1, http://pngu.mgh.harvard.edu/purcell/plink/) [15]

Affymetrix 500K chip SNP genotype data from our previous publication [10] were uploaded into the GenABEL R package. This included 85 vCJD patients and 1481 Wellcome Trust Case–control Consortium (http://www.wtccc.org.uk) samples. ‘QTSCORE’ analysis found those SNPs with lower p-values than those from the prion protein gene and these were removed from subsequent analysis (leaving 287545). These SNPs had already been shown, or presumed, to be false positives by genotyping in our earlier publication [10]. The chromosome data were prephased (haplotyped) using the minimac command line protocol ‘mach1’ with the parameters: Iterations of the Markov sampler to use for haplotyping – 20; Haplotypes to consider during each update – 200; Repeated iterations for when random (but plausible) sets of haplotypes for each individual should be drawn – 5. The MACH operation followed using the recommended command line operations together with the 1000 Genomes (http://www.1000genomes.org) haplotype dataset for the reference SNP data (MACH website file: 2010-06.CEU.map.tgz). Due to computing power limits a subset of the control samples was used for these analyses. Data for all 85 vCJD patients, and 665 of the 1481 controls were used. For the large chromosome 2 only 415 controls were used. This operation generated a total of 6858238 SNPs across the genome to be used for association testing.

### Association

Association testing using the PLINK software suite followed the quality control guidance given by Anderson *et al*[16]. The chromosome specific MACH output files were converted into PED/MAP format files using the Perl script ‘convert_mach.pl’ from GenGen (Imputation Helper; http://www.openbioinformatics.org/gengen), then merged and converted to the PLINK binary format (bim, bed, and fam).

QC Screening

•No individuals had elevated missing data rates (>3%).

•Five individuals (one vCJD patient and four controls) were excluded as they had heterozygosity at more than 3 × standard deviation of the calculated heterozygosity rate.

•The ‘missing rate’ was calculated for each SNP, and with a threshold of >10% failure, 4626404 SNPs were removed from the subsequent analysis.

•The difference in genotype call rates between cases and controls gave a total of 337307 SNPs with p < 0.00001 significance, that were removed.

•PLINK association testing removed those markers that were not in Hardy Weinberg equilibrium (151347), those that fail a genotype ‘missingness test’ (4289134; GENO > 0.1), and those failing a minor allele frequency test (3800790; MAF < 0.01).

•This QC analysis left 1544357 SNPs for the association analysis on 84 vCJD and 661 control samples.

Association testing, with adjustment for multiple testing, using PLINK showed no evidence of population stratification (genomic inflation factor is 1, with mean chi-squared statistic 0.997035).

### Resequencing

The PLCXD3 gene intron 1/exon 2 boundary (847 bp) was amplified by PCR using forward (5’-gtggctcatttgagggagag-3’) and reverse (5’-atttgaggtttccccctgac-3’) primers (Eurofins MWG Operon, UK). PCR products were sequenced using the reverse primer (5’-catttccgcatgagcttttt-3’) and BigDye Terminator v3.1 Cycle Sequencing kit (Invitrogen, UK) on an ABI3130 capillary sequencer.

### Resequencing statistics

Fisher’s Exact Test was used to compare allele and genotype frequencies for the PLCXD3 SNPs.

### Splice site motif analysis

The online tool SVM-BPfinder (http://regulatorygenomics.upf.edu/Software/SVM_BP/) was used to predict the effects of the SNP variation on the recognition sites for the branch point and polypyrimidine tract motifs of the 3’ splice site. The following sequence for the 3’ end of intron 1 was uploaded to the website: 5’-GCAAGAGGGAAAGAAG[K]TGGGAAGGG[M]AAAGACGTGGAAATTAACCACACTATGCCTTGT[W]ATTCTCCTAG-3’ (SNPs in brackets). Eight ‘fasta’ format files were used covering all possible combinations of alleles for the three SNPs.

### Ethical consent

Consent was given for research and the study is covered by approval from the Lothian Health Board, Lothian Research Ethics Committee (reference MCO/103/90). Written informed consent for research was obtained from each patient or a family relative.

### Sample collection

DNA used in this study was extracted from blood samples taken from patients whilst under clinical investigation.

### Ethnic origin

Both vCJD and sCJD populations were sourced from UK resident patients. The majority are Caucasian with three vCJD and three sCJD patient described as non-Caucasian. For control samples, the 1000 Genome results are from individuals of European ancestry and the Exome Variant Server data are from individuals of European American ancestry.

## Results

The initial input data for this study were 287545 Affymetrix 500K chip genotypes [10] and following imputation and QC screening this increased to a total of 1544357 SNPs for PLINK association testing. The most significant SNPs were on chromosome 20 within close proximity of the prion protein gene (*PRNP*) (value p = 1.484e-05). The next most significant region was on chromosome 5 at the PLCXD3 (phosphatidylinositol-specific phospholipase C, X domain containing 3) gene locus with p-values just significant after Bonferroni correction (value p = 0.0236). The next SNPs were on chromosome 9 (Gene: GLIS3 (GLIS family zinc finger 3)), chromosome 12 (near TSPAN8 (tetraspanin 8)), and chromosome 1 (near RGS4 (regulator of G-protein signalling 4)) although these were not significant after Bonferroni correction [see Additional file 1: Table S1].

**Figure 1:**
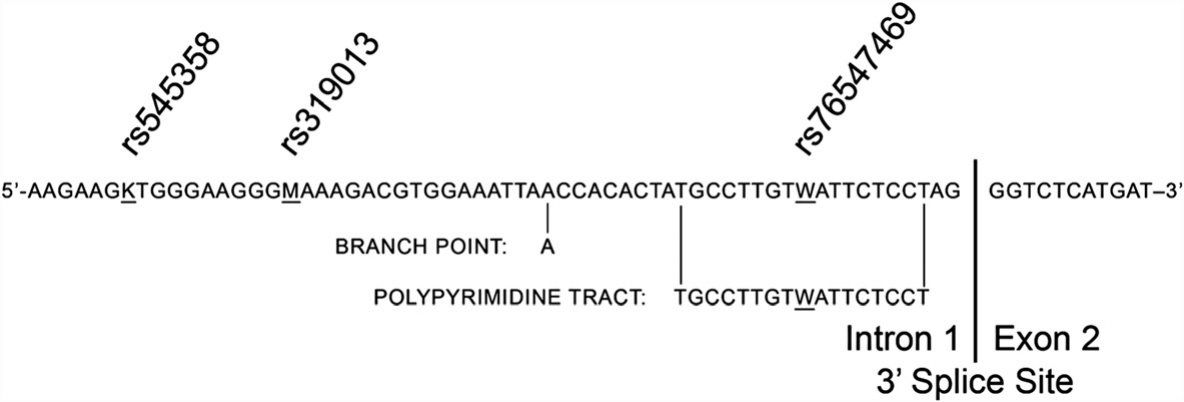
**Diagram showing the DNA sequence from the intron 1**/**exon 2 border of the PLCXD3 gene showing positions of the SNPs under investigation.**

The PLCXD3 gene was chosen for Sanger resequencing and using data from the Exome Variant Server (http://evs.gs.washington.edu/EVS/) we selected a region near the splice junction of intron 1 and exon 2 (genome assembly GRCh37 position Chr5:41382626-41382697) that had two informative SNPs with high minor allele frequencies (rs319013 MAF 35.45%; rs76547469 MAF 5.39%). In close proximity to the two chosen SNPs was a third variant (rs545358) that was not on the Exome Variant Server but was included in subsequent genotype analysis (Figure 1). 120 vCJD and 109 sCJD patients were sequenced and the results compared with European control data from 1000 Genomes and the Exome Variant Server. All three SNPs show statistically significant differences between disease and control samples, and rs545358 had higher risk allele frequencies in vCJD than sCJD (Table 1).

**Table 1:** **Summary of resequencing data with Fisher**’**s test analysis of variant CJD**, **sporadic CJD and controls**

**Genotype Count**						
	rs545358	rs319013	rs76547469			
	GG:GT:TT	CC:CA:AA	TT:TA:AA			
sCJD (n = 109)	15:3:91	48:24:37	99:2:8			
vCJD (n = 120)	49:3:68	52:21:47	110:3:7			
1000 Genomes (n = 379)	1:41:337	n/a	n/a			
Exome Variant Server (n = 4241)	n/a	599:1894:1748	3838:428:17			
Fisher's Test Results for sCJD and vCJD versus Online Controls						
Test	dbSNP Identifier	Genotype/Allele Comparison	p value	Odds Ratio	95% Confidence Interval	Significance^1^
sCJD vs. vCJD						
	rs545358	G vs. T	2.04E-10	0.246	0.151 - 0.393	***
		GG vs. TT	6.46E-06	0.230	0.110 - 0.459	***
		GG vs. GT	0.172	0.312	0.038 - 2.579	
	rs319013	C vs. A	0.5738	1.126	0.767 - 1.655	
		CC vs. AA	0.6568	1.172	0.629 - 2.190	
		CC vs. CA	0.5933	0.809	0.375 - 1.734	
	rs76547469	T vs. A	0.7255	0.847	0.398 - 1.795	
		TT vs. AA	0.7906	0.788	0.234 - 2.589	
		TT vs. TA	1	1.348	0.151 - 16.444	
sCJD vs. Controls						
	rs545358	G vs. T	2.01E-05	2.962	1.769 - 4.923	***
		GG vs. TT	2.69E-09	55.001	8.253 - 2315.479	***
		GG vs. GT	2.30E-10	163.748	17.131 - 8175.872	***
	rs319013	C vs. A	4.69E-08	2.134	1.615 - 2.827	***
		CC vs. AA	4.22E-09	3.783	2.387 - 6.038	***
		CC vs. CA	6.73E-14	6.318	3.758 - 10.882	***
	rs76547469	T vs. A	0.07015	0.633	0.386 - 1.101	
		TT vs. AA	1.61E-07	0.055	0.022 - 0.151	***
		TT vs. TA	0.003525	5.519	1.478 - 46.367	**
vCJD vs. Controls						
	rs545358	G vs. T	< 2.2e-16	12.038	7.957 - 18.471	***
		GG vs. TT	< 2.2e-16	237.889	39.562 - 9180.580	***
		GG vs. GT	< 2.2e-16	530.747	60.122 - 16384.000	***
	rs319013	C vs. A	1.25E-06	1.894	1.452 - 2.471	***
		CC vs. AA	3.56E-08	3.227	2.108 - 4.951	***
		CC vs. CA	< 2.2e-16	7.834	4.593 - 13.812	***
	rs76547469	T vs. A	0.2474	0.748	0.452 - 1.318	
		TT vs. AA	3.67E-06	0.070	0.027 - 0.203	***
		TT vs. TA	0.005828	4.088	1.353 - 20.212	**
Significance	* 0.05 - 0.001	** 0.001 - 0.0001	*** <0.0001			

^1^ Significance: * 0.05-0.001; ** 0.001-0.0001; *** <0.0001.

The online splice site analysis tool (SVM-BPfinder) output predicted that the most suitable polypyrimidine tract splice motif was obtained when ‘T’ was present at SNP rs76547469 (with adenosine present the polypyrimidine tract is not recognised), and with the branch point adenosine at 28 nucleotides from the splice site [see Additional file 2: Table S2].

## Discussion

Three intronic SNPs in the PLCXD3 gene (rs319013, rs76547469, and rs545358) are associated with increased risk of CJD compared with published healthy control data and represent the first major association of a non-*PRNP* candidate gene. These SNPs lie at the junction of intron 1 and exon 2 in close proximity to the splice site motifs. Exon 2 codes for the main active structural domain of PLCXD3 and therefore any alteration to the functioning of the spliceosome at this part of the gene could have a significant impact on the activity of the whole protein. Using the online tool ‘SVM-BPfinder’ we found that with the replacement of thymidine with adenosine at SNP rs76547469 the polypyrimidine tract motif is not recognised and therefore this change is likely to weaken the spliceosome activity. Further investigation into potential PLCXD3 protein structural alterations and the role of the protein in CJD pathology are now needed to clarify the association between the disease phenotype and the presence of these SNP risk alleles. It is proposed that PLCXD3 would have an effect on the pathological pathways of CJD rather than having a direct molecular interaction with the specific misfolding of prion protein.

Phospholipase C X-domain containing proteins (PLCXDs) are a subtype of the phosphatidylinositol-specific phospholipase C (PI-PLC) protein family that is a key component of eukaryotic signal transduction with a role in inositol phospholipid metabolism. PI-PLC enzymes are receptor-regulated phosphodiesterases that control cellular processes by regulation of cytosolic calcium and/or protein kinases. They regulate hormones, growth factors, and neurotransmitters by generating the calcium-regulator inositol-1,4,5-triphopshate (InsP_3_) and the membrane-bound protein kinase C activator diacylglycerol (DAG) [17]. The PI-PLC family has a characteristic pairing of X and Y-domains that together form a barrel-like secondary structure for the catalytic site. In contrast, PLCXD proteins have only the X-domain, as also seen in bacteria. Studies of bacterial PI-PLC proteins indicate that for PLCXD3 the absence of the Y-domain and other protein motifs suggests it has a role in calcium independent phosphatidylinositol metabolic processes with a preference for cleavage of only PI [18]. So far at least three isoforms of PLCXD proteins have been identified: PLCXD1, PLCXD2, and PLCXD3. These are proposed to have distinct functions due to their varied tissue distribution and cellular location but are all likely to be active phosphodiesterases as they increase the turnover of inositol phosphate (InsP) [19]. In a cell culture model PLCXD3 was found in cytoplasmic and perinuclear vesicles and from analysis of RNA levels it was expressed in a wide range of tissue types suggesting a potential key cellular role, with predominant expression seen in the brain [19]. The role of PLCXD3 in neurodegenerative disease is as yet unknown, although PI-PLC and specifically Ca^2+^ homeostasis have been associated with neurodegeneration. Torres *et al*[20] discussed the role of calcium homeostasis and protein folding at the endoplasmic reticulum in prion disease and highlighted a number of relationships between calcium ion levels and the abnormal form of the prion protein. The genetic association seen in our data may provide a novel target to understand these pathways. It is of significant importance that the functional pathways which include PLCXD3 may also involve MTMR7, another phosphatidylinositol phospholipase that was the primary candidate gene from our initial GWA study [10].

## Conclusions

Our data provide the first highly significant genetic association outside the prion protein gene locus for the variant and sporadic forms of CJD. We hope that the PLCXD3 gene locus will now become a focus for more studies in these diseases. To investigate the mechanisms that may exist in both acquired and idiopathic forms of CJD in relation to the PLCXD3 protein we aim to identify the expression level, localisation, and forms of PLCXD3 in disease tissue and non-CJD controls.

## Competing interests

The authors declare that they have no conflicting interest in relation to this study.

## Author’s contributions

MTB designed the project and analysed the data. RSGK supervised the study. MTB, PS-J, and RSGK wrote the manuscript. All authors read and approved the final manuscript.

## Pre-publication history

The pre-publication history for this paper can be accessed here:

http://www.biomedcentral.com/1471-2350/14/91/prepub

## Supplementary Material

Additional file 1: Table S1 Description: PLINK output for the top 30 targets from association analysis and ordered by significance. Click here for file

Additional file 2: Table S2 Description: Scoring of potential splice site motifs for PLCXD3 SNP variants using SVM-BPfinder. Click here for file
